# Photoluminescent Detection of Human T-Lymphoblastic Cells by ZnO Nanorods

**DOI:** 10.3390/molecules25143168

**Published:** 2020-07-10

**Authors:** Alexander Tamashevski, Yuliya Harmaza, Ekaterina Slobozhanina, Roman Viter, Igor Iatsunskyi

**Affiliations:** 1 Institute of Biophysics and Cell Engineering of National Academy of Sciences of Belarus, Akademicheskaya St. 27, Minsk 220072, Belarus; tayzoe@mail.ru (A.T.); garmaza@yandex.com (Y.H.); slobozhanina@ibp.org.by (E.S.); 2 Institute of Atomic Physics and Spectroscopy, University of Latvia, 19, Raina Blvd, 1586 Riga, Latvia; roman.viter@lu.lv; 3 Center for Collective Use of Scientific Equipment, Sumy State University, 31, Sanatornaya str., 40018 Sumy, Ukraine; 4 NanoBioMedical Centre, Adam Mickiewicz University in Poznan, Wszechnicy Piastowskiej str. 3, 61-614 Poznan, Poland

**Keywords:** zinc oxide nanorods, MOLT-4 cell line, T-lymphoblasts detection, cluster of differentiation proteins, monoclonal antibody anti-CD5, room temperature photoluminescence

## Abstract

The precise detection of cancer cells currently remains a global challenge. One-dimensional (1D) semiconductor nanostructures (e.g., ZnO nanorods) have attracted attention due to their potential use in cancer biosensors. In the current study, it was demonstrated that the possibility of a photoluminescent detection of human leukemic T-cells by using a zinc oxide nanorods (ZnO NRs) platform. Monoclonal antibodies (MABs) anti-CD5 against a cluster of differentiation (CD) proteins on the pathologic cell surface have been used as a bioselective layer on the ZnO surface. The optimal concentration of the protein anti-CD5 to form an effective bioselective layer on the ZnO NRs surface was selected. The novel biosensing platforms based on glass/ZnO NRs/anti-CD5 were tested towards the human T-lymphoblast cell line MOLT-4 derived from patients with acute lymphoblastic leukemia. The control tests towards MOLT-4 cells were performed by using the glass/ZnO NRs/anti-IgG2a system as a negative control. It was shown that the photoluminescence signal of the glass/ZnO NRs/anti-CD5 system increased after adsorption of T-lymphoblast MOLT-4 cells on the biosensor surface. The increase in the ZnO NRs photoluminescence intensity correlated with the number of CD5-positive MOLT-4 cells in the investigated population (controlled by using flow cytometry). Perspectives of the developed ZnO platforms as an efficient cancer cell biosensor were discussed.

## 1. Introduction

Nowadays, one of the major challenges is to develop portable and low-cost diagnostic systems for agriculture and medicine. In this regard, it is important to develop novel nanostructures and nanomaterials that could be applied for the design of effective biosensing platforms. One-dimensional (1D) nanostructures such as nanotubes, nanowires, nanorods have attracted attention due to their potential use as building blocks in fabricating nanoscale devices and (bio)sensors. Such nanostructures have many unique properties, such as large surface to volume ratio and biocompatibility, that is extremely important for biosensing applications [1]. One-dimensional metal oxide nanostructures based on ZnO have received great attention for sensing applications because of their simple fabrication procedures, biocompatibility, chemical stability, and significant optical properties, which could be used as a transducer in biosensors development [2,3]. It was demonstrated that zinc oxide-based nanorods (ZnO NRs) are chemically stable and have a high specific surface area, capable of detecting clinically important biomolecules with high sensitivity and reproducibility [2,4].

ZnO is a direct band-gap (E_g_ = 3.37 eV) semiconductor with a large exciton binding energy (60 meV), exhibiting near-UV emission, transparent conductivity, and piezoelectricity [1,5]. Moreover, ZnO is biosafe, biocompatible, and can be used for biomedical applications [4].

Up to now, many scientific works have been focused on the application of ZnO NRs in biosensor technology. Jang et al. showed that ZnO NR systems could be used for the detection of H1N1 swine influenza virus with a sensitivity of 1.0 pg/mL [2]. In another study, an electrochemical immunosensor based on the ZnO NRs matrix electrode for the detection of *Legionella pneumophila* with excellent selectivity and detection limit (1.0 pg/mL) was developed by Park et al. [4]. Sanguino et al. used ZnO nanorod structures deposited on micrometer Au electrodes that function as three-dimensional matrixes, and only then anti-horseradish peroxidase antibodies were immobilized [6]. Such an interdigitated capacitive sensor technology enables the possibility for a simplified detection approach of direct antigen distinguishing in complex biological samples.

There are numerous studies describing the application of ZnO nanostructures for biosensing applications [7,8,9,10]. The application of ZnO NRs photoluminescence for the detection of bioobjects was investigated by Viter et al. in a series of articles [11,12,13]. A novel optical immunosensor for detecting the pathogen Salmonella typhimurium for the first time was introduced [11]. It was found that immobilization of the bioselective layer (anti-Salmonella antibody) to ZnO NRs leads to an increase in the photoluminescence (PL) intensity, and after interaction with Salmonella antigens, the PL intensity decreases proportionally to the antigens concentration. Using photoluminescent ZnO NRs and bovine leukemia virus (BLV) protein gp51, a novel recognition system was developed for the determination of specific antibodies produced in cattle as a humoral immune response against BLV antigens [12]. In work [13], the authors demonstrated a photoluminescence-based immunosensor for the detection of Ochratoxin A, which was tested at a wide range of toxin concentrations from 10^−4^ ng/mL till 20 ng/mL. All these publications indicate that biosensors with an optical transducer (photoluminescence) demonstrate significant sensitivity.

There are various markers associated with different cancer types. Therefore, a lot of research groups make an effort to create biosensors based on ZnO NRs for early-stage cancer detection. For example, a photo-electrochemical immunosensor based on ZnO NR was developed for the detection of metastasis-suppressing protein NDPK-A, which is used as a biomarker for a wide range of cancers [14]. In recent studies [15,16], nanohybrids of ZnO NRs with Au NPs or multiwall carbon nanotubes, respectively, were used as sensitive systems for the specific detection of CA-125—the ovarian cancer antigen. In study [17], the authors presented a ZnO nanowires coated three-dimensional (3D) scaffold chip device for the effective immunocapture and classically visible and colorimetric detection of exosome—cell-derived vesicles that have the potential to be novel biomarkers for noninvasive diagnosis of cancers. In our previous work [18,19], a portable analytic system for cancer cell detection, based on ZnO NRs were reported as well. ZnO NRs were used as biomarkers in solution to recognize cancer cells, using an as ‘up-bottom’ system when the target cells (PA-1; HeLa; HEK-293; Hep-G2 cells) were attached to a glass slide [18], as ‘bottom-up’ approach for pathologic B-cell differential detection (IM-9 suspension cells against donor’s B-lymphocytes), when ZnO NRs form biosensors templated on a glass slide [19]. In this research, the change in the photoluminescence (PL) intensity as a function of IM-9 suspension cells concentration were used as an indicator for the detection of the analyte.

In the current work, we demonstrate the possibility of PL detection of human leukemic cells—T-lymphoblasts (MOLT-4 cell line), using ZnO NR platforms and specialized monoclonal antibodies (MABs) against cluster of differentiation (CD) proteins on the surface of investigated cancer cells (anti-CD5). The suspension cell culture MOLT-4 derived from the peripheral blood of a 19-year-old male with acute lymphoblastic leukemia in relapse was used as the origin of the T lymphoblastic cells.

**Figure 1:**
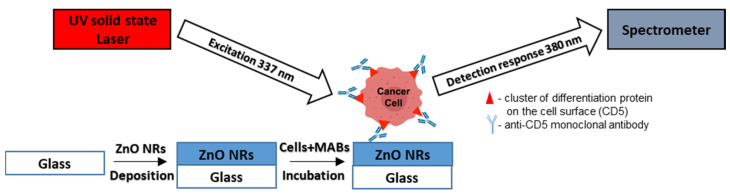
Schematic image of the detection system and the mechanism of T-lymphoblastic cell detection.

Figure 1 represents the schematic illustration of the detection system and the mechanism of cancer cell detection.

## 2. Results and Discussion

### 2.1. Structural Characterization of ZnO Nanorods

**Figure 2:**
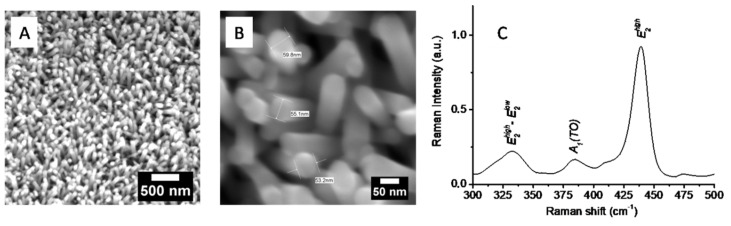
(**A**) SEM, (**B**) high-resolution SEM images, and (**C**) Raman spectrum for as-prepared ZnO NRs.

The microstructure of obtained ZnO NRs deposited on a glass substrate was characterized by SEM. Figure 2a,b display typical SEM images of ZnO NRs. ZnO NRs prepared according to our method are uniform in diameter, length, and crystalline structure. The length of NRs is in the range of 400–700 nm and approximately 50 ± 10 nm in diameter. A detailed SEM characterization of glass/ZnO NRs substrates is reported in Ref. [1]. In order to confirm the crystallinity of obtained NRs, Raman spectroscopy analysis was performed. Figure 2c shows Raman spectrum for ZnO NRs grown on glass substrate. The main peak at 440 cm^−1^ corresponds to E_2_ (low) wurzite mode. The weak peak at 385 cm^−1^ is attributed to A1 (TO) mode, indicating that NRs have perfect crystal quality [2].

### 2.2. CD5 Expression in the Leukemic Cell Line MOLT-4

It is known that surface proteins (antigen) of human lymphocytes, CD5, characterized at a certain stage of their differentiation, were chosen as biomarkers for T-lymphoblastic cells determination [20]. CD5 is a glycoprotein normally found on T cells, and a subset of immunoglobulin M (IgM)-secreting B cells known as B-1a cells, as well as a regulator for B cells [21], but only on a small number of such peripheral blood B cells that are presented in healthy adults. CD5 is not expressed in hematopoietic stem cells and other non-hematopoietic cells. However, it is a specific surface marker expressed in the majority of T-cell malignancies, including T-acute lymphocytic leukemia and T-lymphoma, in addition to some B-cell lymphomas [22]. Besides, T cells express higher levels of CD5 than B cells.

**Figure 3:**
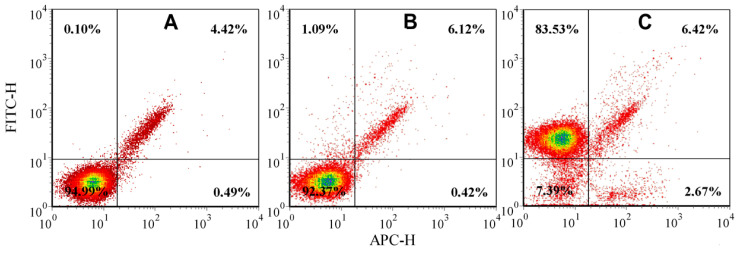
Flow cytometry-based analysis of the T-lymphocytic antigen CD5 in the human T-lymphoblast cell line MOLT-4 where: (**A**–**C**). Flow cytometric quadrant analysis of the MOLT-4 cell line: autofluorescence of cells (**A**), anti-IgG2a-FITC labeled cells (**B**), anti-CD5-FITC labeled cells (**C**). Upper quadrants: IgG2a-positive (**B**) or CD5-positive cells (**C**).

At the first stage, the expression of CD5 antigens on MOLT-4 cells was analyzed by flow cytometry using a mouse anti-human CD5 and IgG2a (isotopic control) monoclonal antibodies conjugated with fluorescein isothiocyanate (FITC). Figure 3 shows the autofluorescence of the gated cells (Figure 3a) and a sample of cells that has been stained with anti-IgG2a-FITC (Figure 3b) and anti-CD5-FITC (Figure 3c) antibodies. It was found that the anti-CD5-FITC antibody had strong and specific binding to 82.74% of MOLT-4 cells using flow cytometric quadrant analysis (Figure 3c), whereas binding of the IgG2a control was low—2.69% (Figure 3b). This numbering (82.74%) refers to the percentage of T-lymphoblastic cells in cultivated MOLT-4 cell lines.

It is known that antibodies can bind to the cells surface in a specific manner, where the Fab part of the antibody binds to a high-affinity specific targetor the Fc part of the antibody binds to the Fc receptors on the surface of cells. However, they can also bind in a non-specific manner, where the Fab portion binds to a low affinity, non-specific target. IgG2a, in our case, is an isotopic control designed to measure the level of non-specific background signal caused by anti-CD5 antibody based upon the tissue type of the sample. This background signal is the result of non-specific immunoglobulin’s binding to Fc receptors present on the T-cell surface.

### 2.3. ZnO NRs Optical Properties after Antibodies Immobilization

As mentioned previously, the PL is used as a transducer signal. The room temperature photoluminescence (RT PL) of bare ZnO NRs sample deposited on a glass substrate was reported [11]. Two peaks were found in the RT PL spectrum—a narrow and intense peak at 380 nm and a wide non-symmetric peak, centered at around 520 nm. The PL of ZnO nanostructures usually exhibits near-band-edge (NBE) emissions and the broad deep-level emission (DLE) corresponding to the UV and visible emission, respectively [3]. We have focused on the NBE emission peak (380 nm) intensity as the most informative signal.

Testing concentrations of monoclonal antibodies were selected as 2.5; 5.0; 12.5; 25.0, and 50.0 µg/mL. It is known that covalent and non-covalent interactions formed with different methodologies are used to functionalize the ZnO nanostructures, introducing different organic and functional groups and enhancing the attachment of biomolecules through electrostatic interactions or specific chemical bonds [3]. The simplest method for surface biofunctionalization of metal oxide nanostructures with biomolecules is physical absorption. Biomolecules may adhere to the surface of the nanostructure because of Van der Waals force, electrostatic forces, hydrophobic interactions, and hydrogen bonding [23,24]. The High isoelectric point (IEP) of ZnO (pH 9.1) is helpful in the immobilization of biomolecules on the ZnO surface. Taking into account that the isoelectric point of ZnO at the pH of 7.0–7.2 is relatively high (IEP~9.5), a possible mechanism of anti-CD5 immobilization on ZnO NRs is based on a strong electrostatic interaction between a positively charged matrix ZnO and a negatively charged group of proteins (antibody) with a lower isoelectric point (IEP~5–6.5) [24].

**Figure 4:**
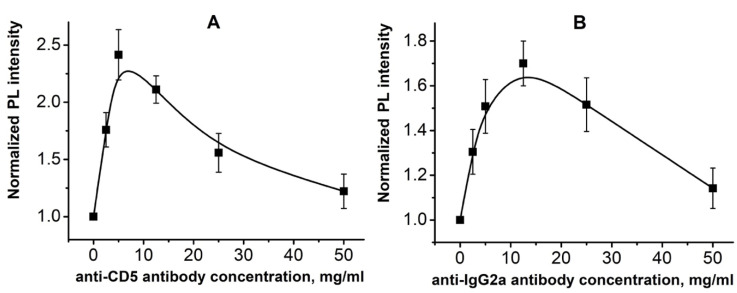
RT PL intensities of glass/ZnO NRs after immobilization of anti-CD5 (**A**) and anti-IgG2a (**B**) monoclonal antibodies in concentration ranges of 2.5–50.0 µg/mL.

Using PL analysis, it was found that anti-CD5 MAB immobilization at concentrations of 2.5 and 5.0 µg/mL on ZnO NRs substrate (glass/ZnO NRs/anti-CD5) leads to a dose-dependent increase in RT PL intensity (Figure 4a). After immobilization of anti-CD5 on glass/ZnO NRs in the range of 12.5–50.0 µg/mL, the RT PL intensity of the obtained biosensing platforms was reduced (Figure 4a). One can conclude that 5.0 µg/mL is an optimal concentration of the monoclonal antibody for T-lymphoblasts detection using ZnO NRs platform because this amount of anti-CD5 MAB provides optimal coverage of the ZnO NRs surface. For comparison, the RT PL of glass/ZnO NRs after immobilization of the isotopic control for anti-CD5 MAB–anti-IgG2a were measured (glass/ZnO NRs/anti-IgG2a). It was shown that the PL intensity versus the anti-IgG2a concentration demonstrates a similar behavior. However, the maximum was reached at 12.5 µg/mL of anti-IgG2a (Figure 4b), while for anti-CD5 MAB, the maximum was observed at around 5.0 µg/mL (Figure 4a). It might be explained by the various number of antibodies immobilized on the ZnO NRs surface that also correlates with the difference in molecular weights of anti-IgG2a (~150 kDa) and anti-CD5 (~67 kDa) MABs [25,26].

The increase of PL is attributed to electrostatic interactions between antibodies and the ZnO NR surface. This interaction depends on the number and the type of immobilized MABs. The observed decrease in RT PL intensity under the immobilization of MABs at higher concentrations could be explained by the formation of double or triple biolayers of MABs and a subsequent crystallization that leads to the PL quenching [19].

### 2.4. MOLT-4 Cell Culture Testing with ZnO NRs Platforms

Flow cytometry measurements showed that the investigated human T-lymphoblast cell line MOLT-4 after cultivation contained 80 to 90% CD5-positive cells and not more than 2–3% of IgG2a-labeled cells from this population.

**Figure 5:**
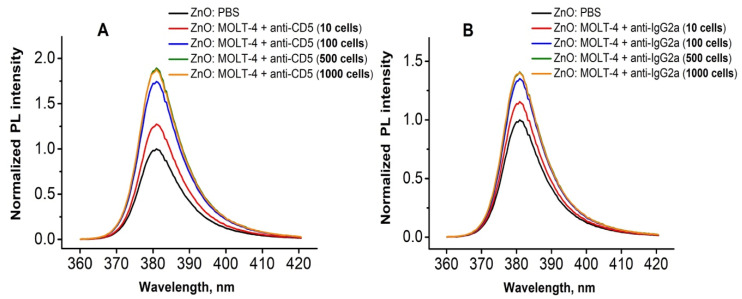
RT PL spectra of ZnO NRs platform after immobilization of the different number of MOLT-4 cells conjugated with anti-CD5 (**A**) and anti-IgG2a (**B**) monoclonal antibodies.

**Figure 6:**
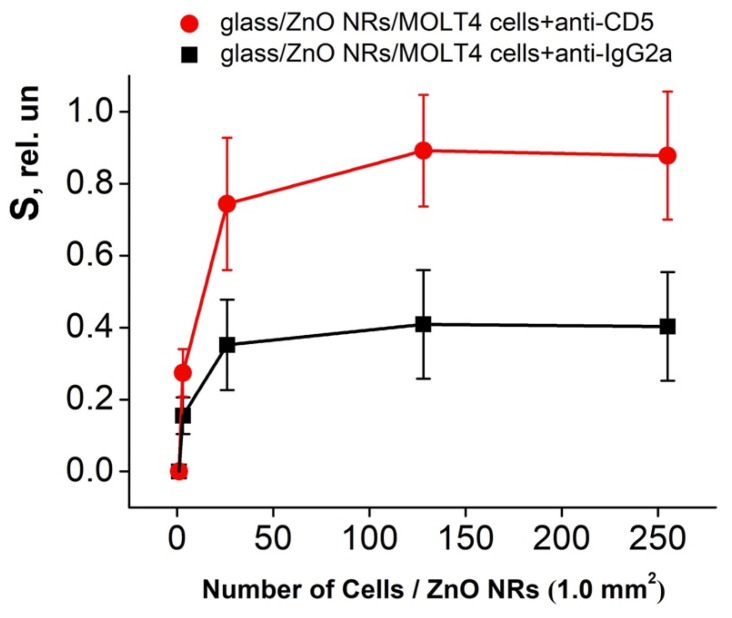
The dynamics of the relative RT PL intensity changes of the ZnO NRs platform after immobilization of the MOLT-4 cells conjugated with anti-CD5 and anti-IgG2a monoclonal antibodies.

The change in the PL signal (in this case, NBE PL peak) of the ZnO NR was used as an indicator for the presence of the analyte. In order to study the sensitivity of the produced biosensing platforms, human T-lymphoblast MOLT-4 cells in a wide range of concentrations (from 10 till 1000 cells for one sample) were used (Figure 5 and Figure 6). PL biosensing tests were performed by using MOLT-4 cells pre-treated with anti-CD5 and anti-IgG2a antibodies. Figure 5a demonstrates the increase in the ZnO PL intensity with the rise of the amount of CD5-positive cells in the investigated populations. However, one may observe the saturation of PL signal at higher concentrations of MOLT-4 cells (500–1000 cells). A similar tendency of PL signal was also observed for cells conjugated with anti-IgG2a (Figure 5b), which characterizes the non-specific background signal caused by the CD5 MABs. Such changes in the PL signal by adding the analyte (e.g., antigens, cells) is also observed in studies reported by Myndrul et al. [27,28], and by Viter et al. [12,13]. It was attributed to the surface modification caused by the antigen-antibody reaction, which leads to changes in the charge carriers concentration and their subsequent radiative recombination, and as a consequence, changes in the PL intensity.

Figure 6 shows the relative PL signal changes after the immobilization of the human MOLT-4 cells conjugated with anti-CD5 and anti-IgG2a antibodies. The calculation of cells was represented on a 1.0 mm^2^ surface of ZnO NRs platform. The relative PL intensity (S, the sensitivity) obtained by the interaction of ZnO NRs and MOLT-4 cells conjugated with anti-CD5 and anti-IgG2a antibodies was calculated according to equation 1 [12,13]:

(1) $$S=\frac{I-I_{0}}{I_{0}}$$

where I_0_ and I are PL intensities before and after deposition of MOLT-4 cells conjugated with anti-CD5 and anti-IgG2a antibodies, respectively.

It was found that the “glass/ZnO NRs/MOLT-4 cells+anti-CD5” system finds 3 to 128 T-lymphoblast cells per 1.0 mm^2^ of ZnO NR platforms with an increase in the S value on average from 0.27 to 0.89 (Figure 6). However, using the “glass/ZnO NRs/MOLT-4 cells+anti-IgG2a” construction as a negative control, a response of PL signal was also obtained. In this case, the S value increased on average from 0.16 to 0.41 with a rise in the T-lymphoblast cells concentration from 3 to 128 per 1.0 mm^2^ of ZnO NRs (Figure 6). This S value (calculated for the “glass/ZnO NRs/MOLT-4 cells+anti-IgG2a”) was used as an initial point for the estimation of the “net effect” in the glass/ZnO NRs platform response. Thus, one can see (Figure 6) that this effect was averaged from 1.7 times (3 cells per 1.0 mm^2^) to 2.2 times (128 cells per 1.0 mm^2^) relative to platforms with a negative control.

In our previous work [19], another leukemic cell line—IM-9 (B-lymphoblastoid cells)—was investigated, and the mechanism of adhesion of B-cells conjugated with anti-CD19 and anti-IgG1 antibodies to glass/ZnO NRs substrate was proposed. Based on the analysis of both cell lines, it was concluded that antibodies localized on the surface of T-lymphoblasts as globules result in an increase in the free cell surface area and a number of additional links with ZnO NRs. Besides, additional Raman peaks were observed after immobilization of the complex B-cell+anti-CD19 antibody, which corresponds to vibrations of amino and carboxyl groups [19]. In order to explain the changes of PL intensity of ZnO NRs after immobilization of MOLT-4 cells pre-treated with anti-CD5 antibodies (Figure 5), one may propose the mechanism based mostly on the charge rearrangement at the surface of ZnO when the biorecognition events occur, which could be attributed either to a decrease in the negative surface potentials in ZnO [29] or an increase in the number of newly formed surface Zn-S bonds [30]. However, the real mechanism of PL changes after the immobilization of biological analytes is still under investigation.

Despite the promising results obtained in this report, further studies are needed to optimize the detection at low and high concentrations in order to determine the response curve, which should allow us to estimate the detection limit and the sensitivity of the sensor.

## 3. Materials and Methods

### 3.1. Chemical Reagents

All the chemicals in the experiment were used without further purification. Trypan blue, RPMI-1640 medium, fetal calf serum, l-glutamine, penicillin, streptomycin, l-butanol, ethanol, were purchased from (Sigma-Aldrich, Steinheim, Germany). FITC-conjugated mouse anti-human monoclonal antibody CD5, FITC-conjugated mouse anti-human monoclonal antibody IgG2a, mouse anti-human monoclonal antibody CD5, mouse anti-human monoclonal antibody IgG2a were purchased from (Immunotech, Marseille, France). Cell line MOLT-4 (ATCC^®^ CRL-1582™) was obtained from ECACC (Oxford, UK).

### 3.2. Flow Cytometry Analysis of Cell Surface Marker CD5 Expression in the Leukemic Cell Line

Human MOLT-4 cell line (T lymphoblasts; acute lymphoblastic leukemia) were cultured in a complete RPMI-1640 medium supplemented with 10% (*v*/*v*) heat-inactivated fetal calf serum (FC) (56 °C for 60 min), l-glutamine (2 mmol/L), penicillin (100 U/mL), streptomycin (100 µg/mL) at 37 °C in a 5% CO_2_: 95% air humidified atmosphere and harvested at the log phase of growth. A cell culture maintains between 3–9 × 100.000 cells/mL in 5% CO_2_; 37 °C. Cell viability was determined by Trypan blue dye exclusion.

Cell culture MOLT-4 was analyzed for CD5-positive T-lymphoblast cells by flow cytometry. Cell suspensions (1 × 10^6^ cells/mL) were stained for surface marker expression using fluorescein isothiocyanate (FITC)-conjugated mouse anti-human CD5 antibody and mouse anti-human IgG2a isotype control of CD5 antibody for 1 h at room temperature in the dark and washed twice with PBS 300 × g for 10 min. Cells were analyzed using FACSCanto II (BD Biosciences, San Jose, CA, USA). For analysis, forward and side scatter gates were set to include viable cells and to exclude dead cells and debris. A minimum of 20,000 lymphocyte-gated events was acquired on flow cytometry, with data analyzed by FCSExpress Version 3 Research Edition De Novo software for enhanced acquisition analysis.

### 3.3. ZnO Nanorods Deposition for Platform Preparation and Its Structural Properties

ZnO nanorods (ZnO NRs) were obtained by the gaseous-disperse synthesis. For further use of ZnO NRs, they were dispersed in 99.8% l-butanol in order to prepare 1 mg/mL alcohol solution, and then ultrasonically treated for 30 min, 44 KHz. Eighteen millimeter square glass coverslips (substrates) were cleaned in 70% ethanol and deionized water and treated by the use of plasma technology in an O_2_ atmosphere using Plasma Cleaner (Harrick Plasma, Ithaca, NY, USA). After that, 10 µl of ZnO NRs stock solution was dropped on a glass substrate and dried at room temperature for 12 h. ZnO NRs formed a layer on the substrate (glass/ZnO NRs), which was further annealed at 300 °C in air atmosphere for 2 h. The crystallinity of the obtained ZnO NRs based platforms (glass/ZnO NRs) were studied by Raman spectroscopy (Renishaw micro-Raman spectrometer equipped with a confocal microscope Leica). The microstructures of the obtained ZnO NRs based platforms (glass/ZnO NRs) were studied by scanning electron microscopy (SEM, Hitachi, Tokyo, Japan). In this way, glass/ZnO NRs platform was prepared for the modification by antibodies and cells.

### 3.4. Modification of ZnO NRs Based Platform by Antibody and Antibody-Pre-Treated Cells

Anti-CD5 antibody was used for the immobilization on the glass/ZnO NRs substrate in order to form glass/ZnO NRs/anti-CD5 structures. For this, 4.0 µL of anti-CD5 antibody at the final concentration of 2.5–50.0 µg/mL was dropped on ZnO nanorod substrates and maintained overnight on cold.

Human MOLT-4 cells were incubated in vitro with two types of antibodies: anti-CD5 or anti-IgG2a, for 1 h at room temperature. The received conjugates of MOLT-4 cells+anti-CD5 and MOLT-4 cells+anti-IgG2a were dropped on the ZnO nanorod substrates and maintained overnight on cold. These samples were washed with PBS pH 7.2 to remove cells unbounded to ZnO nanorods. Only dry samples were analyzed.

### 3.5. Optical Characterization of ZnO NRs Based Platform

Characterization of optical properties was performed for the following structures: glass/ZnO-NRs/anti-CD5, glass/ZnO-NRs/MOLT-4 cells+anti-CD5 and glass/ZnO-NRs/MOLT-4 cells+anti-IgG2a.

Photoluminescence (PL) spectra of ZnO nanorod platforms were detected similarly, as described in [12,13]. PL was excited using UV solid-state laser LGI-21 (output power 0.4 mW, with 337 nm excitation wavelength). The emission spectra were recorded at room temperature in the range of 370–800 nm by 1 nm steps using spectrometer HR2000+ (Ocean Optics, Dunedin, FL, USA). The measurement parameters were integration time—0.2 s (seven repeats).

## 4. Conclusions

In conclusion, this study demonstrates that the photoluminescent properties of monoclonal antibodies-targeted ZnO NRs could be used for the development of a biosensing platform towards human leukemic T-cells (e.g., MOLT-4 leukemic cell line) detection. It was shown that T-lymphoblast cells bind to CD5-targeted ZnO NRs platforms with high selectivity and the PL signal significantly increased in comparison with the signal from the IgG2a targeted platforms (control sample). An increase of the ZnO NRs photoluminescence intensity correlated with the number of CD5-positive cells in the investigated populations that was controlled by the flow cytometry experiments. It was demonstrated that human MOLT-4 cells conjugated with anti-CD5 monoclonal antibodies even at extremely low cell concentrations, from 3 to 128 cells per 1.0 mm^2^, could be detected by the ZnO NRs platform. The obtained results demonstrate the suitability of the RT PL of ZnO NRs to be employed in the development of biosensors for the detection of other relevant analytes.
